# Addition of intraperitoneal cisplatin and etoposide to first-line chemotherapy for advanced ovarian cancer: a randomised, phase 2 trial

**DOI:** 10.1038/s41416-018-0036-7

**Published:** 2018-06-14

**Authors:** Tingyan Shi, Rong Jiang, Jinjin Yu, Huijuan Yang, Dongsheng Tu, Zhiyuan Dai, Yang Shen, Yuqin Zhang, Xi Cheng, Huixun Jia, Ruiqin Tu, Huaying Wang, Jie Tang, Yuting Luan, Shumo Cai, Rongyu Zang, for the SGOG-OV/AICE Investigators

**Affiliations:** 1 Ovarian Cancer Program, Division of Gynecology Oncology, Department of Obstetrics and Gynecology, Zhongshan Hospital, Fudan University, Shanghai, China; 2 Department of Gynecologic Oncology, Fudan University Cancer Hospital, Shanghai, China; 3 Department of Obstetrics and Gynecology, Wuxi Cancer Hospital, Wuxi, China; 4 Department of Mathematics and Statistics, Queen’s University, Kingston, ON Canada; 5 Department of Obstetrics and Gynecology, Suzhou Municipal Hospital, Suzhou, China; 6 Department of Obstetrics and Gynecology, Zhongda Hospital Southeast University, Nanjing, China; 7 Clinical Statistics Center, Fudan University Cancer Hospital, Shanghai, China

**Keywords:** Ovarian cancer, Chemotherapy

## Abstract

**Background:**

We assessed the efficacy of adding intraperitoneal (IP) chemotherapy to standard first-line intravenous (IV) chemotherapy in epithelial ovarian cancer (EOC) patients.

**Methods:**

Patients with stage IIIC-IV EOC who underwent optimal debulking surgery were randomly assigned to four cycles of weekly IP chemotherapy with cisplatin (50 mg/m^2^) and etoposide (100 mg/m^2^) followed by six cycles of IV chemotherapy every 3 weeks (IP/IV arm), or were administered IV chemotherapy alone (IV arm). The primary endpoint for this study was the 12-month non-progression rate (NPR).

**Results:**

Between 4/2009 and 9/2015, 218 patients were randomised, of whom 215 initiated treatment. In the IP/IV arm, 90.6% of patients completed 4 cycles of IP chemotherapy. The 12-month NPRs were 81.9% and 64.2% in the IP/IV and IV groups, respectively (HR 0.48 (95% CI 0.27–0.82)). The median progression-free survival (PFS) was increased in the IP/IV arm compared with that in the IV arm (22.4 vs. 16.8 months; HR 0.66 (0.48–0.91)) and in a subgroup with no gross cytoreduction (31.1 vs. 16.8 months; HR 0.46 (0.26–0.82)). Similar findings were detected with regard to time to first subsequent anticancer therapy (TFST) (25.9 vs. 18.0 months; *P* = 0.009) and time to second subsequent anticancer therapy (TSST) (40.8 vs. 30.1 months; *P* = 0.042). Grade 3/4 leukopenia, anaemia and gastrointestinal events were more common in the IP/IV arm, but the treatment burden was considered acceptable.

**Conclusions:**

IP chemotherapy prior to IV chemotherapy was associated with an increased 12-month NPR and a longer TSST than IV alone in patients with EOC, albeit with acceptable toxic effects. Long-term follow-up is warranted to identify the effects of IP therapy on overall survival.

## Introduction

Three randomised phase 3 studies have demonstrated that cisplatin-based intraperitoneal (IP) chemotherapy is an effective management for patients with epithelial ovarian cancer (EOC) who underwent primary optimal cytoreduction^1–3^. A median 16-month improvement in overall survival (OS) was associated with cisplatin IP treatment in GOG-172^3^. Long-term follow up data showed a 23% reduction in the risk of death associated with combined IP/ intravenous (IV) chemotherapy versus IV chemotherapy alone^4^. The advantage of IP/IV over IV platinum-based chemotherapy were highlighted in the “Clinical Announcement” by the National Cancer Institute (NCI) in 2006^5^. However, to date, IP chemotherapy is far less utilised as a standard primary management paradigm in clinical practice^6^ due to its disadvantages: higher incidence of toxicities; catheter-related complications; lower completion rate due to the inconvenience of IP administration; as well as the absence of a well-accepted optimal regimen^7^. Researchers should take into consideration a balance between IP therapeutic benefits and disadvantages.

We previously reported a weekly IP chemotherapy regimen comprising cisplatin and etoposide that had been used extensively in our institution and other Chinese centres over the past three decades^8^. In contrast to other reported treatment models of IP combined with IV synchronously^9^, in our study, patients with advanced EOC received sequential chemotherapy (weekly IP chemotherapy followed by standard front-line IV chemotherapy) and obtained a survival benefit with acceptable complications and toxicities^8^.

We chose to combine etoposide with cisplatin as first-line IP chemotherapy for several reasons. First, etoposide was once considered a primary therapy for EOC^8,10,11^. Second, in a pharmacokinetic study of IP cisplatin and etoposide, the free (non-protein bound) etoposide peritoneal exposure was 65-fold greater than that in plasma^12^, indicating pharmacological evidence for the regional IP use of etoposide. Subsequently, at the University of California, IP cisplatin and etoposide treatment has been demonstrated to be effective in EOC^10,11^, consistent with our previous study^8^. Third, our data showed that the toxicities associated with sequential IP therapy were acceptable. Fourth, etoposide is inexpensive and has well been accepted in developing countries.

In view of the long-term clinical practice, we designed a prospective, randomised controlled trial to investigate the survival benefit of intraperitoneal cisplatin and etoposide (AICE) as first-line therapeutic drugs in an IP regimen.

## Materials and methods

### Study design and patients

This investigator-initiated, phase 2, AICE trial was designed by Shanghai Gynecologic Oncology Group (SGOG, www.ShanghaiGOG.org) and performed by SGOG centres in China. Patients with optimal debulking surgery were randomly assigned postoperatively in a 1:1 ratio to receive either IP cisplatin 50 mg/m^2^ and etoposide 100 mg/m^2^ weekly for 4 cycles followed by IV carboplatin AUC 5 and paclitaxel 175 mg/m^2^ or docetaxel 60–75 mg/m^2^ every 3 weeks for 6 cycles (IP/IV arm, research arm) or IV carboplatin AUC 5 and paclitaxel 175 mg/m^2^ or docetaxel 60–75 mg/m^2^ every 3 weeks for 6 cycles (IV arm, control arm). A randomisation code was computer generated by an independent statistician. Randomisation was performed by a centralised office with patient data screened by the principal investigator.

Patients with optimal debulked stage IIIC and IV primary epithelial ovarian, fallopian tube, or peritoneal cancer, excluding lymph node metastasis, were enrolled in this trial. We defined a complete resection of R0 strictly as en bloc resection according to the Fudan Standard that was previously described in recurrent settings^13–15^. As a result, our measurement of R0 is more conservative than that in other published studies. Specifically, when all carcinomatosis are cytoreduced to no gross residual by electronic devices, if these diseases are NOT resected by an en bloc approach, we defined it as residual disease (RD) ≤0.5 cm. Additional eligibility criteria are described in Supplementary Methods.

All cases were centrally reviewed to confirm patients’ surgical and pathological eligibility for enrollment. Although without a strictly blinded review, pathological reports, operative documents and eligibility information were collected before registration. Quality controls included data source verification by monitoring, double data entry and in-house monitoring in the central study office.

The study was performed in accordance with the Declaration of Helsinki. All patients provided written informed consent before participation. The study protocol was approved by the ethics committee of Fudan University Cancer Hospital in 18 April 2009 (IRB number: 090371-2), and the first patient was enrolled in 26 April 2009. This trial is registered with ClinicalTrials.gov’s number NCT01669226.

### Assessment of adverse events

In the IP/IV arm, the IP cisplatin or etoposide was delivered in 0.5 liters of normal saline. A total of 1.5–2 L of normal saline was infused through a peritoneal catheter that was implanted during cytoreductive surgery.

Eligible patients who received at least one cycle of IP or IV chemotherapy were assessed for toxic effects. Adverse events were graded according to the CTCAE v3.0. If toxicities could not be tolerated, investigators conventionally reduced the chemotherapeutic dosage or changed the regimen. According to the CTCAE evaluation criteria, in cases of grade 4 neutropenia with temperature >38.5 °C, prolonged grade 4 neutropenia (persisting ≥7 days), or grade 4 thrombocytopenia, the dose of paclitaxel or docetaxel should be decreased by 25% and carboplatin by 1 AUC unit. In the case of grade 1–3 bone marrow suppression, treatment would be administered according to the specific symptoms, and chemotherapy would be delayed if necessary. Every effort was made to maintain the planned schedule.

### Endpoints

The primary endpoint was the 12-month non-progression rate (NPR). After completion of first-line therapy, patients were followed every 3 months over the first 5 years, and then, every 6 months thereafter. Disease status was assessed by patient symptoms, physical examination and imaging at the end of treatment (after six cycles of IV chemotherapy, or if protocol treatment stopped prematurely for any reason). Recurrence was diagnosed by one or more of the following: physical examination; elevated CA125 levels as defined by the Gynecologic Oncology Intergroup^16–18^; and radiological imaging, including ultrasound, computed tomography scan (CT)/magnetic resonance imaging (MRI) or positron emission tomography (PET)/CT scan.

Secondary endpoints included progression-free survival (PFS), completion rate and toxicity of IP chemotherapy, and OS. PFS was defined as the time from the date of randomisation to the diagnosis of the first recurrence or last follow-up, whichever came first. Time to first subsequent anticancer therapy (TFST) and time to second subsequent anticancer therapy (TSST) were defined as the time from the date of randomisation to the date of first and secondary recurrent anticancer therapy, respectively.

### Statistical analyses

In this study, IP chemotherapy was utilised as a first-line therapy in patients with stage III EOC. However, before starting our trial, we did not find any survival data of IP chemotherapy in patients with either stage III or IV disease. There is an iPOCC trial (JGOG 3019) including stage II–IV patients; however, that study is still ongoing. Therefore, based on clinical practice and preliminary data from Fudan University^19^, we planned to recruit 200 patients who would be randomised to the IP/IV and IV arms at a 1:1 ratio with a type I error rate of 0.1 and a power of 80%, considering 10% loss to follow-up, and anticipating an 18% increase (from 48% to 66%) of the 12-month NPR in the IP/IV arm. The sample size calculation was performed using the PASS software program (version 11.0, NCSS, LLC, 329 North 1000 East Kaysville, Utah 84037, USA).

Eligible patients were analysed according to the intention-to-treat principle. Survival analyses were performed when the last subject completed 1 year of follow-up. The primary outcome measure of NPR was analysed using the *χ*^2^-test. The *χ*^2^ or Mann–Whitney *U*-tests were used to compare the differences of baseline characteristics and adverse events between the two groups. Median survival was evaluated using the Kaplan–Meier method, and differences were determined using the log-rank test. Hazards ratios (HRs) and confidence intervals (CIs) were estimated with the Cox proportional hazards regression model. We performed subgroup analyses to investigate whether residual disease had any effect on the survival benefit of IP chemotherapy. All statistical analyses were performed using SPSS software (version 16.0, The Predictive Analytics Company, Chicago, USA). A two-sided *P* value of <0.05 was considered significant.

## Results

**Fig. 1:**
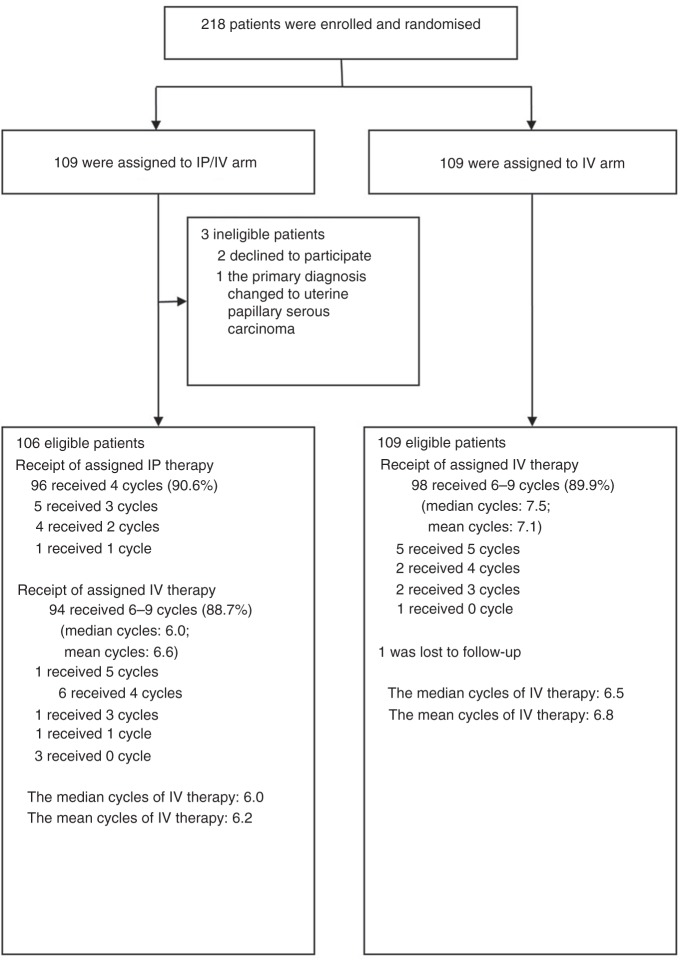
Trial profile

Between 26 April 2009 and 7 September 2015, we screened 220 patients; 218 of whom were enrolled in the study (Fig. 1). Of the 218 eligible patients, 166 were from Fudan University Cancer Hospital, 44 were from Zhongshan Hospital, Fudan University, six were from Wuxi Cancer Hospital, one was from Suzhou Municipal Hospital, and one from Zhongda Hospital Southeast University. From the total patient pool, 109 were assigned to the IP/IV arm and 109 to the IV arm. Three patients in the IP/IV arm were ineligible and withdrawn from the trial: two declined to participate, and one had inadequate pathology. Of the 215 patients, baseline characteristics were well balanced between the two treatment arms (Table 1). R0 was achieved in 35.8% and 36.7% of the patients in the IP/IV arm and the IV arm, respectively. The median cycles of standard IV therapy was 6 and 7.5 cycles in IP/IV arm and in IV arm, respectively; the median first-line treatment time after randomisation were similar in these two arms, with 5.3 and 5.1 months correspondingly.

**Table 1:** Baseline characteristics

Characteristic	IP/IV arm (*N* = 106)	IV arm (*N = *109)	*P* value^a^
Median age (range)	54 years	57 years	0.517
(35–78)	(40–75)		
FIGO stage			0.706
Stage IIIC	83 (78.3%)	83 (76.1%)	
Stage IV	23 (21.7%)	26 (23.9%)	
Primary tumour			0.336
Epithelial ovarian cancer	96 (90.6%)	99 (90.8%)	
Fallopian tube cancer	9 (8.5%)	6 (5.5%)	
Primary peritoneal cancer	1 (0.9%)	4 (3.7%)	
Histology			0.537
Serous	99 (93.4%)	101 (92.7%)	
Endometrioid	1 (0.9%)	2 (1.8%)	
Clear cell	1 (0.9%)	4 (3.7%)	
Undifferentiated	4 (3.8%)	2 (1.8%)	
Others	1 (0.9%)	0 (0%)	
Grade			0.773
Low	2 (1.9%)	3 (2.8%)	
High	104 (98.1%)	104 (95.4%)	
NA	0 (0%)	2 (1.8%)	
ECOG performance status			0.580
0	44 (41.5%)	40 (36.7%)	
1	58 (54.7%)	62 (56.9%)	
2	4 (3.8%)	7 (6.4%)	
ASA Score			1.000
1	62 (58.5%)	63 (57.8%)	
2	43 (40.6%)	46 (41.3%)	
3	1 (0.9%)	1 (0.9%)	
Preoperative CA125 measurement			0.946
Median serum level	870.5 U/ml	928.0 U/ml	
(range)	(13.6–25210.9)	(47.1–72090.3)	
Ascites			0.186
Median volume	1000 ml	800 ml	
(range)	(0–8000)	(0–7000)	
Neoadjuvant chemotherapy			0.171
Yes	14 (13.2%)	22 (20.2%)	
No	92 (86.8%)	87 (79.8%)	
Residual disease in the whole abdomen			0.601
0 cm	38 (35.8%)	40 (36.7%)	
0.1–0.5 cm	56 (52.8%)	52 (47.7%)	
0.5–1 cm	12 (11.3%)	17 (15.6%)	

*ASA* American Society of Anesthesiologists, *ECOG* Eastern Cooperative Oncology Group, *FIGO* International Federation of Gynecology and Obstetrics, *NA* not available.

^a^ Tested by *χ*^2^ or Mann–Whitney *U*-test

Of the 106 eligible patients assigned to the IP/IV arm, 96 patients (90.6%) completed 4 cycles of the planned IP chemotherapy, and 94 patients (88.7%) received at least 6 cycles of assigned IV therapy (Fig. 1). Ten patients (9.4%) did not complete the assigned IP therapy for the following reasons: renal dysfunction (one patient); chemical peritonitis (one patient); catheter-associated abdominal infection (one patient); leakage of the intraperitoneal fluid around the catheter exit site (one patient); grade 3 or 4 bone marrow depression (four patients); and grade 3 gastrointestinal event (two patients). Of the 109 patients assigned to the IV arm, 98 patients (89.9%) received at least 6 cycles of assigned IV therapy. One patient (0.5%) was lost to follow-up, and the chemotherapy data were not available (Fig. 1).

All adverse events and grades are listed in Supplementary Table S1. As shown in Table 2, grade 3 and 4 leukopenia (53.8% vs. 35.2%), anaemia (23.6% vs. 5.6%) and gastrointestinal events (10.4% vs. 1.9%) were more common in the IP/IV arm than in the IV arm (*P* = 0.006, *P* < 0.001 and *P* = 0.010, respectively). One patient in the IP/IV arm died of gastrointestinal bleeding due to grade 4 thrombocytopenia seven months after completing 4 cycles of IP followed by 4 cycles of IV therapy. Another patient in the IP/IV arm died of mesenteric venous thrombosis 12 days after completing 4 cycles of IP followed by 4 cycles of IV therapy.

**Table 2:** Grade 3 or 4 Adverse Events

Adverse Events	IP/IV arm (*N* = 106)^a^	IV arm (*N* = 107)^b^	*P* value^c^
No. (%)			
Leukopenia	57 (53.8)	38 (35.2)	0.006
Neutropenia	70 (66.0)	64 (59.3)	0.305
anaemia	25 (23.6)	6 (5.6)	<0.001
Platelet count < 50*10^9	13 (12.3)	8 (7.5)	0.241
Gastrointestinal event	11 (10.4)	2 (1.9)	0.010
Infection	12 (11.3)	5 (4.7)	0.073
Thromboembolic event (Grade 5)	1 (0.9)^d^	0 (0)	0.498

^a^ 3 patients in IP group did not receive IV chemotherapy.

^b^ 1 patients did not receive any protocol-based therapy. 1 patient only received 3 cycles of IV therapy after cytoreduction and the data of adverse events were missed.

^c^*P* values were calculated by *χ*^2^-test (grades 0, 1, and 2 vs. grades 3 and 4).

^d^ 1 patient died of mesenteric venous thrombosis after completing 4 cycles of IP therapy and 4 cycles of IV therapy. In the intraperitoneal chemotherapy group, other 3 patients encountered grade 2 thromboembolic events (deep venous thrombosis of the lower extremities). And in the intravenous chemotherapy group, 1 patient encountered a grade 2 thromboembolic event (Upper-extremity deep vein thrombosis), which was PICC line-associated thrombosis^31^

To better manage patients, prevent catheter-related infection, record adverse events, etc., patients in the IP/IV arm received all cycles of IP therapy during hospitalisation. All patients received IV chemotherapy in the outpatient department. The mean length of hospitalisation was 43.1 days and 20.7 days in the IP/IV and IV arms, respectively. The mean inpatient cost was CNY 64,180.8 (equal to $9,338.2) in the IP/IV arm, with only CNY 11,778.9 (equal to $1695.1) in the IV arm (CNY 52,401.9, equal to $7,424.4). Despite a significant difference between these two arms (*P* = 0.002), the costs are comparable.

**Fig. 2:**
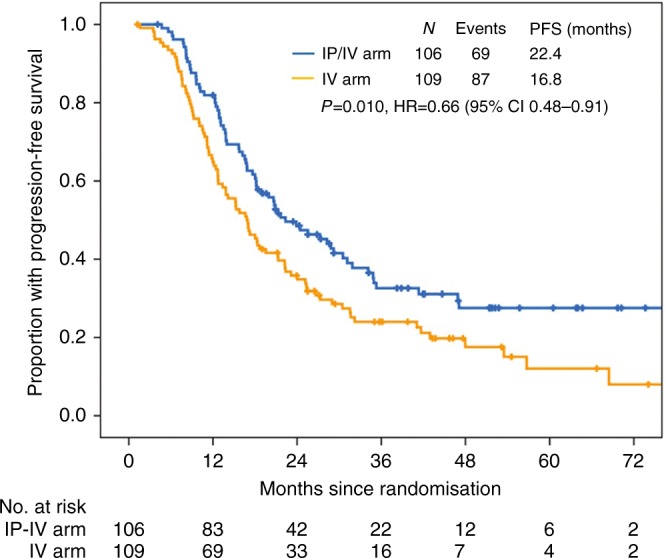
Kaplan–Meier distribution of progression-free survival time. Patients in the IP/IV arm had improved PFS compared with those in the IV arm (*P* = 0.010; HR = 0.66; 95% CI, 0.48–0.91)

Patient follow-up was censored on March 28, 2017 (1.5 years after the last patient enrolled). One patient died of mesenteric venous thrombosis 4.1 months after randomisation, and another died of gastrointestinal bleeding due to grade 4 thrombocytopenia 12.0 months after randomisation. Both cases were censored. With a median follow-up of 50.0 months (95% CI 45.7-54.2), the overall 12-month NPR was 72.6% (156/215) with 81.9% (86/105) and 64.2% (70/109) in the IP/IV and IV arms, respectively (hazards ratio (HR) 0.48 (95% CI 0.27–0.82); *P* = 0.005). The median PFS was 22.4 months (95% CI 15.6–29.1) in the IP/IV arm and 16.8 months (95% CI 13.3–20.3) in the IV arm (*P* = 0.010; HR = 0.66; 95% CI, 0.48–0.91; Fig. 2). Analysis of OS has been kept blinded due to data immaturity. However, we evaluated TFST and TSST as clinically meaningful extensions of PFS. Similar findings were detected in TFST (25.9 (95% CI 19.3–32.6) vs. 18.0 months (95% CI 14.8–21.2); *P* = 0.009; HR = 0.65; 95% CI, 0.47–0.90), and TSST (40.8 (95% CI 28.1–53.6) vs. 30.1 months (95% CI 25.0–35.2); *P* = 0.042; HR = 0.68; 95% CI, 0.47–0.99).

**Fig. 3:**
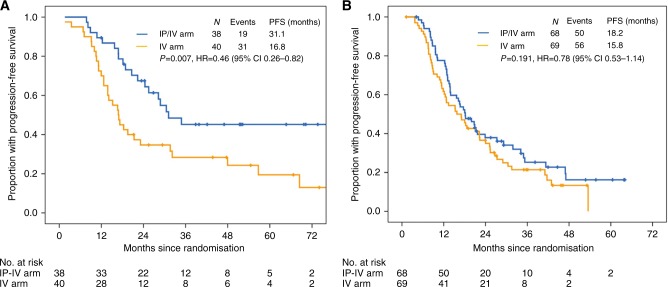
Kaplan–Meier distribution of progression-free survival time in subgroup of patients with R0 resection (A), and of patients with residual disease 0.1–1 cm (B) in the whole abdomen after primary cytoreductive surgery

Subgroup analyses by RD showed that patients with R0 in the IP/IV arm had a significantly longer PFS than those in the IV arm [31.1 vs. 16.8 months; *P* = 0.007; HR = 0.46; 95% CI, 0.26–0.82) (Fig. 3a). However, in patients with RDs of 0.1–1 cm, we did not observe such significant difference in PFS between these two arms (18.2 vs. 15.8 months; *P* = 0.191; HR = 0.78; 95% CI, 0.53–1.14) (Fig. 3b). PFS benefit from complete resection was observed in the IP/IV arm but not in the IV arm (*P* = 0.003 and 0.248; HR = 0.46 and 0.75; 95% CI 0.27–0.78 and 0.47–1.18, respectively; Supplementary Fig. S1).

## Discussion

Our study confirms a practical and acceptable 28-day dose-dense weekly IP regimen with cisplatin and etoposide followed by standard IV therapy, showing that IP/IV treatment has a survival advantage over IV alone in 12-month NPR, PFS, TFST and TSST. Recently, in animal models, a drug-release microdevice for IP cisplatin was verified to be effective but less toxic, due to its continuous delivery and stabilised drug concentration^20^, further highlighting the relatively dose-dense IP chemotherapy. In addition, out of 36 patients who received neoadjuvant chemotherapy (NACT), there were 6/14 (42.9%) and 8/22 (36.4%) patients who received IP therapy alone or IP combined with IV synchronously as NACT regimens in IP/IV arm and in IV arm, respectively (*P* = 0.697). IP/IV therapy showed a better PFS than IV alone in both NACT and non-NACT subgroups (*P* = 0.044 and 0.084, respectively; unpublished Figure). Our independent weekly IP care is likely to be a dose-dense schedule, maintaining the advantage of a weekly management, in terms of efficacy and overall tolerability.

The findings of this trial were that IP prior to standard IV chemotherapy was associated with improved 12-month NPR of 17.7% than that with IV alone, and a 5.6-month improvement of PFS was observed in the IP/IV arm, which was consistent with results of the GOG-172 study (5.5 months)^3^. It also corresponded to our hypothesis of 18% increase in 12-month NPR. To date, 90 (41.9%) patients in this study have died from tumour disease, a less-than-powerful number to calculate OS. However, more importantly, the current study demonstrated a median 7.9-month TFST and 10.7-month TSST benefit with IP/IV therapy.

In the subset analysis, the greatest survival benefit was achieved in the group of patients with R0 after primary surgery (PFS, 31.1 to 16.8 months, in IP/IV to IV, *P* = 0.007, HR = 0.46). A data analysis of GOG-114 and GOG-172 found that patients with RD 0.1–1 cm could benefit from IP chemotherapy, albeit with 1.89-fold increased risk of death compared with patients with R0^4^. Similarly, Chi et al. recommended an advantage of primary debulking surgery followed by IV/IP chemotherapy in younger patients with RD 0.1–1 cm^21^. The differences might be due to our more conservative and strict standard of R0^13–15^, in which all visible disease should be completely resected by an en bloc approach, not just cauterisation by electronic devices alone. Different from other reports of IP combined with IV synchronously^9^, the current study adopted a sequential chemotherapy administration protocol. Thus, in the R0 subgroup, patients may benefit substantially more from the 28-day dose-dense IP chemotherapy than the R0.1–1 cm subgroup. Another explanation may be that this early postoperative additional IP has more efficiency of chemo-cytoreduction in the R0 rather than the R0.1–1 cm subgroup. This study is still ongoing and requires further follow-up time to yield mature OS data.

In terms of FIGO stage, although no significant difference was observed, we did find a trend for increased PFS in the IP/IV arm for patients with stage IV disease (Supplementary Fig. S2). Recently, Jamieson et al.^22^ reported that the most common site of first and subsequent recurrence in stage IV disease was the abdomen, with only a small number of recurrences arising at extra-abdominal sites. Thus, we posit that ovarian cancer patients with stage IV disease may benefit from IP chemotherapy by decreasing recurrent disease in the abdomen.

The hesitancy to use IP therapy is likely due to higher toxicity, inconvenience, and the risk for catheter-related complications^2,3,7^. In the current study, the IP catheter was placed approximately one month after surgery. Consequently, 90.6% (96/106) of patients completed four cycles of the planned IP chemotherapy, which is substantially higher than other reported IP-related clinical trials. Only three patients failed to receive the assigned IP therapy due to catheter-related complications. It is known that severe thrombocytopenia is a fatal adverse event among haematological toxicities. Our study showed a lower rate of grade 3 and 4 thrombocytopenia than previously reported phase 3 trials^2,3,7^, which demonstrated that the combination use of etoposide with cisplatin did not extra increase the haematological toxicities. Only one patient in the IP/IV arm died of gastrointestinal bleeding due to grade 4 thrombocytopenia. However, this adverse event occurred during the subsequent IV therapy (7.33 months after completing four cycles of IP therapy followed by four cycles of IV therapy). The other patient in the IP/IV arm died of mesenteric venous thrombosis three months after completing four cycles of IP therapy, and it was considered unrelated to the IP therapy. Indeed, this patient was at a high risk for thrombosis but did not receive anticoagulation therapy during the subsequent IV therapy in the local hospital.

Recently, targeted therapy and immunotherapy have become very popular for the management of advanced EOC^23–29^. Novel agents such as bevacizumab and olaparib have been approved as a part of first- and/or second-line management in the United States and Europe. However, considering cost-effectiveness, carboplatin and taxane chemotherapy are still the standard of care in developing countries. Based on the ICON7 study, the estimated cost of maintenance bevacizumab was $3,225 per cycle for 12 cycles. In the subset of patients with high-risk stage IIIC (RD >1 cm) or stage IV EOC with survival benefit (improvements of 3.6 months in PFS and 8.0 months in OS) from bevacizumab, the incremental cost was ~$170,000^25,26,30^, causing a heavy financial burden in developing countries. In the current study, the mean inpatient cost was only CNY 11,778.9 (equal to $1695.1) higher in the IP/IV arm than in the IV arm, and a 5.6-month improvement in PFS was observed. Compared with the cost of bevacizumab, the financial burden on patients who received IP/IV therapy was significantly lower and could be well accepted in developing countries.

This study has several limitations. First, this study lacks phase 3 extended data, albeit with an obvious survival benefit of sequential weekly IP therapy. Second, carboplatin was not considered as IP therapy. Third, more efforts are needed to confirm the survival benefit of etoposide as a first-line therapy for EOC. We have evidence from precision medicine to support the rationale for using etoposide. In the next-generation sequencing technique to test chemotherapeutic sensitivities in tumour tissues after primary debulking surgery, we found that out of four advanced ovarian cancer patients, three (75%) who carried the MDR1 gene mutation were predicted to be sensitive to etoposide (unpublished data). Although lacking large sample sizes, this finding indicates that many patients could benefit from etoposide during first-line therapy.

In summary, the SGOG AICE study shows that addition of IP chemotherapy to standard IV chemotherapy is associated with a higher 12-month NPR and a lengthened TSST compared with IV alone, albeit with added acceptable toxic effects. Compared with targeted therapy, IP/IV therapy has an obviously lower financial burden and a higher rate of completion, which is more practical for patients in developing countries. Long-term follow-up is still ongoing, and a mature OS estimation is expected in future analyses.

## Electronic supplementary material

Supplementary Methods

Supplementary Table S1

Supplementary Figure S1

Supplementary Figure S2
