# Supplementary material for: Addition of intraperitoneal cisplatin and etoposide to first-line chemotherapy for advanced ovarian cancer: a randomised, phase 2 trial
Source: Br J Cancer. 2018 Jun 14;119(1):12–8. doi: 10.1038/s41416-018-0036-7 (PMC6035193; doi:10.1038/s41416-018-0036-7)
Supplement: Supplementary file 1 — Supplementary Methods [file 41416_2018_36_MOESM1_ESM.docx]

**Supplementary Methods**

Additional eligibility criteria were as follows: age ≥18 years to ≤75 years; available to receive IP chemotherapy 5-10 days postoperative or no more than 14 days postoperative for those with bowel resection; Eastern Cooperative Oncology Group (ECOG) performance status of 0-2; no more than three cycles of chemotherapy prior to surgery; adequate bone marrow (absolute neutrophil count greater than 1,500/mm^3^; Platelet count greater than 100,000/mm^3^) and adequate renal (creatinine less than 1.6 mg/dL or creatinine clearance greater than 40 mL/min) and hepatic function (bilirubin less than 1.25 times upper limit of normal (ULN), SGPT less than 2 times ULN); compliance with IP chemotherapy and follow-up.

Given that the study design for IP therapy was sequential and not synchronous with standard IV chemotherapy, we recruited patients who received no more than 3 cycles of neoadjuvant chemotherapy. The cycles of neoadjuvant chemotherapy were included into adjuvant chemotherapy.

Randomization for each patient was performed after primary debulking surgery and before any adjuvant chemotherapy.
