# Supplementary material for: Addition of intraperitoneal cisplatin and etoposide to first-line chemotherapy for advanced ovarian cancer: a randomised, phase 2 trial
Source: Br J Cancer. 2018 Jun 14;119(1):12–8. doi: 10.1038/s41416-018-0036-7 (PMC6035193; doi:10.1038/s41416-018-0036-7)
Supplement: Supplementary file 2 — Supplementary Table S1 [file 41416_2018_36_MOESM2_ESM.docx]

**Supplementary Table S1.** Adverse Events according to treatment groups

| Adverse Events | IP/IV arm  (*N* =106) ^a^ | | | | |  | IV arm  (*N* = 107) ^b^ | | | | | |
| --- | --- | --- | --- | --- | --- | --- | --- | --- | --- | --- | --- | --- |
|  | Grade of severity, N (%) | | | | |  | Grade of severity, N (%) | | | | | |
|  | 0 | 1 | 2 | 3 | 4 |  | 0 | 1 | 2 | 3 | 4 |  |
| Leukopenia | 1 (0.9) | 8 (7.5) | 40 (37.7) | 44 (41.5) | 13 (12.3) |  | 3 (2.8) | 13 (12.0) | 54 (50.0) | 33 (30.6) | 5 (4.6) |  |
| Neutropenia | 1 (0.9) | 5 (4.7) | 30 (28.3) | 40 (37.7) | 30 (28.3) |  | 3 (2.8) | 11 (10.2) | 30 (27.8) | 40 (37.0) | 24 (22.2) |  |
| Anemia | 3 (2.8) | 11 (10.4) | 67 (63.2) | 25 (23.6) | - |  | 24 (22.4) | 34 (31.8) | 43 (40.2) | 5 (4.7) | 1 (0.9) |  |
| Platelet count <50*10^9 | 45 (42.5) | 31 (29.2) | 17 (16.0) | 9 (8.5) | 4 (3.8) |  | 68 (63.6) | 24 (22.4) | 7 (6.5) | 5 (4.7) | 3 (2.8) |  |
| Gastrointestinal event | 4 (3.8) | 64 (60.4) | 27 (25.5) | 11 (10.4) | - |  | 13 (12.1) | 82 (76.6) | 10 (9.3) | 2 (1.9) | - |  |
| Infection | 91 (85.8) | 1 (0.9) | 2 (1.9) | 11 (10.4) | 1 (0.9) |  | 102 (95.3) | - | - | 5 (4.7) | - |  |
| Thromboembolic event | 103 (97.2) | - | 2 (1.9) | - | 1 (0.9)^*^ |  | 106 (99.1) | - | 1 (0.9) | - | - |  |

* This was a grade 5 adverse event. The patient died of mesenteric venous thrombosis after completing 4 cycles of IP therapy and 4 cycles of IV therapy.
